# Absence of high-grade cervical intraepithelial neoplasia in conization specimens from patients with colposcopic biopsy-confirmed high-grade cervical intraepithelial neoplasia: Retrospective study of 1695 cases

**DOI:** 10.3389/fonc.2022.980884

**Published:** 2022-09-14

**Authors:** Yulin Guo, Ying Wang, Qiuzi Peng, Lu Li, Miao Zou, Chaonan Wang, Xufeng Wu, Quanfu Ma

**Affiliations:** ^1^ Department of Gynecologic Oncology, Maternal and Child Health Hospital of Hubei Province, Wuhan, China; ^2^ Hubei Clinical Medical Research Center for Gynecologic Malignancy, Wuhan, China

**Keywords:** conization, absence of high-grade cervical intraepithelial neoplasia, relative factors, follow up, persistence and recurrence rate

## Abstract

Few studies have investigated the absence of high-grade cervical intraepithelial neoplasia (CIN) in excised specimens, and sample sizes of these studies were limited. This study retrospectively analyzed clinical characteristics of 1695 patients with CIN 2/3 to determine the incidence rate and relative factors of CIN 1 or less in conization specimens from patients with colposcopic biopsy-confirmed CIN 2/3. The study group comprised 430 cases of CIN 1 or less in conization specimens, and the control group comprised 1142 cases with high-grade CIN lesions in conization specimens. Univariate and multivariate logistic regression models were established to evaluate relative factors. The 1–9 years follow-up data were analyzed to determine the persistence/recurrence rate. Multivariate logistic regression showed that patients aged 18–24 years (OR (95% CI) = 2.224 (1.014, 4.877)); with a negative hrHPV test result (OR (95% CI) = 3.210 (1.627, 6.331)); a cytology test result of normal (OR (95% CI) = 5.184 (3.138, 8.563)), ASC-US (OR (95% CI) = 3.420 (2.102, 5.564)), LSIL (OR (95% CI) = 2.588 (1.475, 4.541)), or ASC-H (OR (95% CI) = 2.434 (1.306, 4.539)); an indication of CIN 2 on biopsy (OR (95% CI) = 2.290 (1.694, 3.096)), and no glandular involvement (OR (95% CI) = 1.616 (1.205, 2.169)) were more likely to have an absence of high-grade dysplasia in conization specimens. There was no difference in the persistence/recurrence rate between the two groups (x2 = 1.55, P = 0.46). An age of 18–24 years, a negative hrHPV test result, a non-HSIL cytology test result, an indication of CIN 2 on biopsy, and no glandular involvement were relative factors for an absence of high-grade dysplasia in conization specimens. For patients with relative factors, especially young women, informed follow-up should be considered.

## Introduction

High-grade cervical intraepithelial neoplasia (CIN), a lesion caused by infection with high-risk human papillomavirus (hrHPV) and a precursor of uterine cervix carcinoma, is a common health problem among women (1, 2). It is known that the early detection and treatment of high-grade CIN prevents the development of cervical cancer (3, 4). Conization using the loop electrosurgical excision procedure (LEEP) and cold knife conization (CKC) have been accepted as the main techniques for the treatment of high-grade CIN (5, 6). Although conization is a safe and widespread technique, several studies have highlighted its potential complications, including bleeding, infection, incompetent cervix, and cervical stenosis. These complications may result in an increased risk of future problems with pregnancy (7, 8). Moreover, we have encountered excised specimens from patients with high-grade CIN confirmed by colposcopic biopsy that revealed only a grade of CIN 1 or less. This situation not only raises concerns regarding possible misdiagnosis or overtreatment but also creates uncertainty with regard to the appropriate steps to take during follow-up (9–11).

Few studies have investigated the absence of high-grade CIN in excised specimens, and the sample sizes of these studies were limited. Moreover, there were some inconsistencies in the conclusions of these studies (12–14), and the clinical significance of this phenomenon has not been determined. This study retrospectively analyzed the clinical characteristics of 1695 patients diagnosed with CIN 2/3 in the Hubei Maternal and Child Health Hospital and Hubei Clinical Medical Research Center for Gynecologic Malignancy in the past 9 years. This study aimed to determine the rate of occurrence of CIN 1 or less in conization specimens from patients with colposcopic biopsy-confirmed CIN 2/3 and to investigate the relative factors. In addition, results obtained at follow-up after 1–9 years were also analyzed to examine the rates of persistence and recurrence.

## Materials and methods

### Study subjects

A total of 1839 conizations for colposcopic biopsy-confirmed CIN 2/3 were performed in the Hubei Maternal and Child Health Hospital and Hubei Clinical Medical Research Center for Gynecologic Malignancy from January 2010 to December 2019. Patients with prior excision procedures, cervical lesions, or missing key variables were excluded. Cases were diagnosed by cytology and/or hrHPV testing, colposcopy, and biopsy in a three-step diagnostic procedure. The indications for colposcopy-guided biopsy were mainly abnormal cervical cytology, a positive result of hrHPV, a suspicious medical history, or findings on gynecological examination such as postcoital bleeding or an irregular cervical contour.

According to the inclusion and exclusion criteria, 1695 patients were selected for this study. After conization, pathological findings suggested 112 cases of invasive cervical cancer, 11 cases of adenocarcinoma *in situ* (AIS), 1142 cases of CIN 2/3, and 430 cases of CIN 1 or chronic cervicitis. The 112 cases of invasive cervical cancer and 11 cases of AIS were excluded. The 1142 cases of CIN 2/3 were defined as the control group, and the 430 cases of CIN 1 or chronic cervicitis were defined as the study group.

### HrHPV and cytology testing

hrHPV testing in our center was performed using the Cervista™ hrHPV test (Hologic Inc., Madison, WI, USA) and the Digene Hybrid Capture 2 test (Qiagen, Gaithersburg, MD, USA). The Cervista™ hrHPV test is an *in vitro* diagnostic test for the detection of DNA from 14 types of hrHPV (16, 18, 31, 33, 35, 39, 45, 51, 52, 56, 58, 59, 66, and 68). The results were divided into the A9, A7, and A5/6 groups. The Digene Hybrid Capture 2 test detects 13 oncogenic genotypes (16, 18, 31, 33, 35, 39, 45, 51, 52, 56, 58, 59, and 68). The results were classified as positive at a relative light unit (RLU)/cutoff value of ≥ 1 pg/mL. The Kaipu HPV 21 typing test (Kaipu Biotechnology Co., Ltd, Guangzhou, China) was used in most of the referred cases. The HPV 21 classification included 15 high-risk types (HPV 16, 18, 31, 33, 35, 39, 45, 51, 52, 53, 56, 58, 59, 66, and 68) and six low-risk types (HPV 6, 11, 42, 43, 44, and cp8304).

The cytology testing in our center comprised liquid-based cytology testing using the ThinPrep^®^ 2000 system (Hologic, Bedford, MA, USA). Final cytological diagnosis was achieved using the Bethesda system (15). Positive cytology findings included atypical squamous cells of unknown signifcance (ASC-US); atypical squamous cells, cannot exclude high-grade lesion (ASC-H); low-grade squamous intraepithelial lesion (LSIL); high-grade squamous intraepithelial lesion (HSIL); atypical glandular cells (AGC); and AIS.

### Colposcopy and histopathological examination

Normal saline, 5% acetic acid, and 5% iodine were administered to act on the cervix in order to identify the type of transformation zone (TZ) and abnormal colposcopy findings and to perform colposcopically directed biopsy. In the case of a type III TZ, multipoint biopsy and/or endocervical curettage was used in accordance with the medical history, cytology test results, and hrHPV test results.

Pathological diagnosis was performed by two designated specialist pathologists at the same time. In cases of disagreement, the director of the pathology department organized a discussion. Biopsy specimens were analyzed according to the World Health Organization criteria and were classified as negative, CIN 1, CIN 2, CIN 3, or microinvasive carcinoma (16). Some cases of CIN 2/3 were referred from other hospitals. Basic information was obtained by viewing the outpatient medical records. These cases required continuous consultation and confirmation by two designated pathologists before admission.

### Conization procedure and specimen processing

The width and depth of conization were determined according to the type of TZ and the extent of abnormal colposcopy findings. During LEEP conization, the cervix was fully exposed, and a 1.8 cm × 1.8 cm or 1.5 cm × 1.5 cm electrosurgical scalpel was used at the 12 o’clock position on the cervix, which was used as the cutting point, to completely remove the specimens. The base of the conization was subjected to hemostasis by electrocoagulation. In CKC, the suture mark was made at the 12 o’clock position on the cervix, and the suture was used for hemostasis. The procedure was performed by two designated senior physicians. After conization, pathologists cut the conical specimens into multiple tissue blocks, which were then embedded in paraffin and taken for H&E staining and light microscopy observation. All specimens were reviewed with special attention to the status of the margins and glandular involvement.

### Follow-up

Post-conization follow-up was performed at 3, 6, 9, 12, 18, and 24 months—and annually thereafter. Cervical cytology and hrHPV tests were carried out at each visit, and referrals for colposcopy-guided biopsy were indicated for patients with abnormal cytology findings or those who were hrHPV positive. Some patients who were re-examined at local hospitals were followed up by telephone. All the results during the follow-up were recorded.

Persistent/recurrent CIN 2/3 was diagnosed on the basis of a histological diagnosis of CIN 2/3 or repeated cytology results indicating CIN 2/3 (at least two cytology tests). The presence of a histological diagnosis of CIN 1, a cytology result indicating ASC or LSIL, a single cytology finding of HSIL without histological confirmation, or a positive hrHPV test result with negative cytology and/or histology results during follow-up was considered as an indication of persistent/recurrent CIN 1. All patients with a negative cytology test result, a negative hrHPV test result, and, if available, a negative histology test result were considered as having no persistent/recurrent disease. After 2 consecutive years of negative results, these patients were followed up once a year (14).

### Data analysis

Data were analyzed with SPSS 20.0 software(SPSS, Inc, Chicago, IL,USA). Univariate and multivariate logistic regression models were employed to evaluate the relative factors for an absence of high-grade CIN. The variables included age, chief complaint, hrHPV test result, cytology test result, colposcopy biopsy result, glandular involvement, maternal history, and the interval time between biopsy and conization. The odds ratio (OR) and 95% confidence interval (CI) were calculated as estimates of the correlations, and a value of p < 0.05 was considered to be statistically significant in a two-tailed test.

## Results

### Basic information

The average age of the 1572 patients was 38.76 ± 9.84 years. The basic characteristics of the 1572 patients with CIN 2/3 are shown in **Table 1**. The median interval time between biopsy and conization of the 1572 patients was 16 days, P_25_ = 8 days, P_75_ = 27 days. The patients were divided into three groups according to the length of the interval time. Among them, there were 1272 patients with an interval time ≤ 30 days, accounting for 80.92%; 264 patients with an interval time between 31 to 90 days, accounting for 16.79%; and only 36 patients with an interval time ≥ 91 days, accounting for 2.29%.

**Table 1:** Patient characteristics (N = 1572).

Item	N	%
Chief complaint		
Physical examination	1118	71.12%
Postcoital/vaginal bleeding	152	9.67%
Abnormal vaginal discharge	256	16.28%
Others	46	2.93%
hrHPV test result		
Negative	43	3.50%
Positive	1184	96.50%
Cytology test result		
Normal	276	18.84%
ASC-US	472	32.22%
LSIL	226	15.43%
ASC-H	145	9.90%
AGC	7	0.48%
HSIL	334	22.80%
SCC	5	0.34%
Punch biopsy findings		
CIN 2	425	27.03%
CIN 3	1147	72.97%
Pregnancy		
0	115	7.32%
≥ 1	1457	92.68%
Parity		
0	274	17.43%
≥ 1	1298	82.57%
Glandular involvement		
No	853	54.26%
Yes	719	45.74%
Time between biopsy and conization (days)		
≤ 30	1272	80.92%
31–90	264	16.79%
≥ 91	36	2.29%
Conization method		
LEEP	1003	63.80%
CKC	569	36.20%
Conization results		
Negative	337	21.44%
CIN 1	93	5.92%
CIN 2	198	12.59%
CIN 3	944	60.05%
Margins		
Negative	1433	91.16%
CIN 1	59	3.75%
CIN 2/3	80	5.09%
Hysterectomy		
No	1302	82.82%
Yes	270	17.18%

hrHPV, high-risk human papillomavirus; ASC-US, atypical squamous cells of unknown significance; LSIL, low-grade squamous intraepithelial lesion; ASC-H, atypical squamous cells, cannot exclude high-grade lesion; AGC, atypical glandular cells; HSIL, high-grade squamous intraepithelial lesion; SCC, squamous cell carcinoma; LEEP, loop electrosurgical excision procedure; CKC, cold knife conization.

### Risk factors for absence of high-grade dysplasia in conization specimens

**Table 2** shows the logistic regression outcomes for the relative factors for an absence of high-grade dysplasia in conization specimens. Both univariate logistic regression and multivariate logistic regression revealed that an absence of high-grade dysplasia in conization specimens was significantly correlated with age, hrHPV test result, cytology test result, punch biopsy findings, and glandular involvement. Patients with an age of 18–24 years (OR (95% CI) = 2.224 (1.014, 4.877)), a negative hrHPV test result (OR (95% CI) = 3.210 (1.627, 6.331)), a cytology test result of normal (OR (95% CI) = 5.184 (3.138, 8.563)), ASC-US (OR (95% CI) = 3.420 (2.102, 5.564)), LSIL (OR (95% CI) = 2.588 (1.475, 4.541)), or ASC-H (OR (95% CI) = 2.434 (1.306, 4.539)), an indication of CIN 2 on pre-conization biopsy (OR (95% CI) = 2.290 (1.694, 3.096)), and no glandular involvement [OR (95% CI) = 1.616 (1.205, 2.169)] were more likely to have an absence of high-grade dysplasia in conization specimens.

**Table 2:** Logistic regression analysis (OR and 95% CI) for absence of high-grade dysplasia in conization specimens.

Item	Conization result N (%)		Crude OR (95% CI)	Adjusted OR (95% CI)
	Negative/CIN 1	CIN 2/3			
Age				
≤ 24	43 (10.00)	36 (3.15)	3.280 (1.923, 5.593)**	2.224 (1.014, 4.877)*
25–29	75 (17.44)	139 (12.17)	1.482 (0.984, 2.231)	1.628 (0.928, 2.855)
30–34	86 (20.00)	219 (19.18)	1.078 (0.731, 1.590)	1.449 (0.878, 2.391)
35–39	66 (15.35)	206 (18.04)	0.880 (0.586, 1.322)	1.082 (0.645, 1.813)
40–44	51 (11.86)	224 (19.61)	0.625 (0.408, 0.957)*	0.777 (0.452, 1.338)
45–49	50 (11.63)	156 (13.66)	0.880 (0.569, 1.361)	1.077 (0.616, 1.882)
≥ 50	59 (13.72)	162 (14.19)	Ref.	Ref.
Chief complaint				
Postcoital/vaginal bleeding	57 (13.26)	199 (17.43)	0.750 (0.543, 1.035)	
Abnormal vaginal discharge	47 (10.93)	105 (9.19)	1.172 (0.811, 1.693)	
Others	17 (3.95)	29 (2.54)	1.535 (0.832, 2.833)	
Physical examination	309 (71.86)	809 (70.84)	Ref.	
hrHPV test result				
Negative	21 (5.75)	22 (2.55)	2.331 (1.265, 4.294)**	3.210 (1.627, 6.331)**
Positive	344 (94.25)	840 (97.45)	Ref.	Ref.
Cytology test result				
Normal	116 (29.74)	160 (14.88)	6.297 (4.122, 9.619)**	5.184 (3.138, 8.563)**
ASC-US	147 (37.69)	325 (30.23)	3.929 (2.632, 5.863)**	3.420 (2.102, 5.564)**
LSIL	60 (15.38)	166 (15.44)	3.139 (1.986, 4.962)**	2.588 (1.475, 4.541)**
ASC-H	31 (7.95)	114 (10.60)	2.362 (1.391, 4.009)**	2.434 (1.306, 4.539)**
AGC	1 (0.26)	6 (0.56)	1.448 (0.169, 12.374)*	1.454 (0.149, 14.204)
HSIL and above	35 (8.97)	304 (28.28)	Ref.	Ref.
Punch biopsy findings				
CIN 2	187 (43.29)	238 (20.84)	2.923 (2.303, 3.709)**	2.290 (1.694, 3.096)**
CIN 3	243 (56.51)	904 (71.16)	Ref.	Ref.
Glandular involvement				
No	278 (64.65)	575 (50.35)	1.804 (1.434, 2.268)**	1.616 (1.205, 2.169)**
Yes	152 (35.35)	567 (49.65)	Ref.	Ref.
Pregnancy				
0	46 (10.70)	69 (6.04)	1.863 (1.260, 2.754)**	0.861 (0.457, 1.620)
≥1	384 (89.30)	1073 (93.96)	Ref.	Ref.
Parity				
0	103 (23.95)	171 (14.97)	1.789 (1.359, 2.354)**	1.339 (0.803, 2.231)
≥1	327 (76.05)	971 (85.03)	Ref.	Ref.
Time between biopsy and conization (days)				
≤ 30	326 (75.81)	946 (82.84)	Ref.	Ref.
31–90	89 (20.70)	175 (15.32)	1.476 (1.110, 1.962)**	0.995 (0.690, 1.435)
≥ 91	15 (3.49)	21 (1.84)	2.073 (1.056, 4.069)*	1.374 (0.635, 2.972)

*p < 0.05, **p < 0.01; OR, odds ratio; CI, confidence interval.

### Follow-up and results

**Figure 1:**
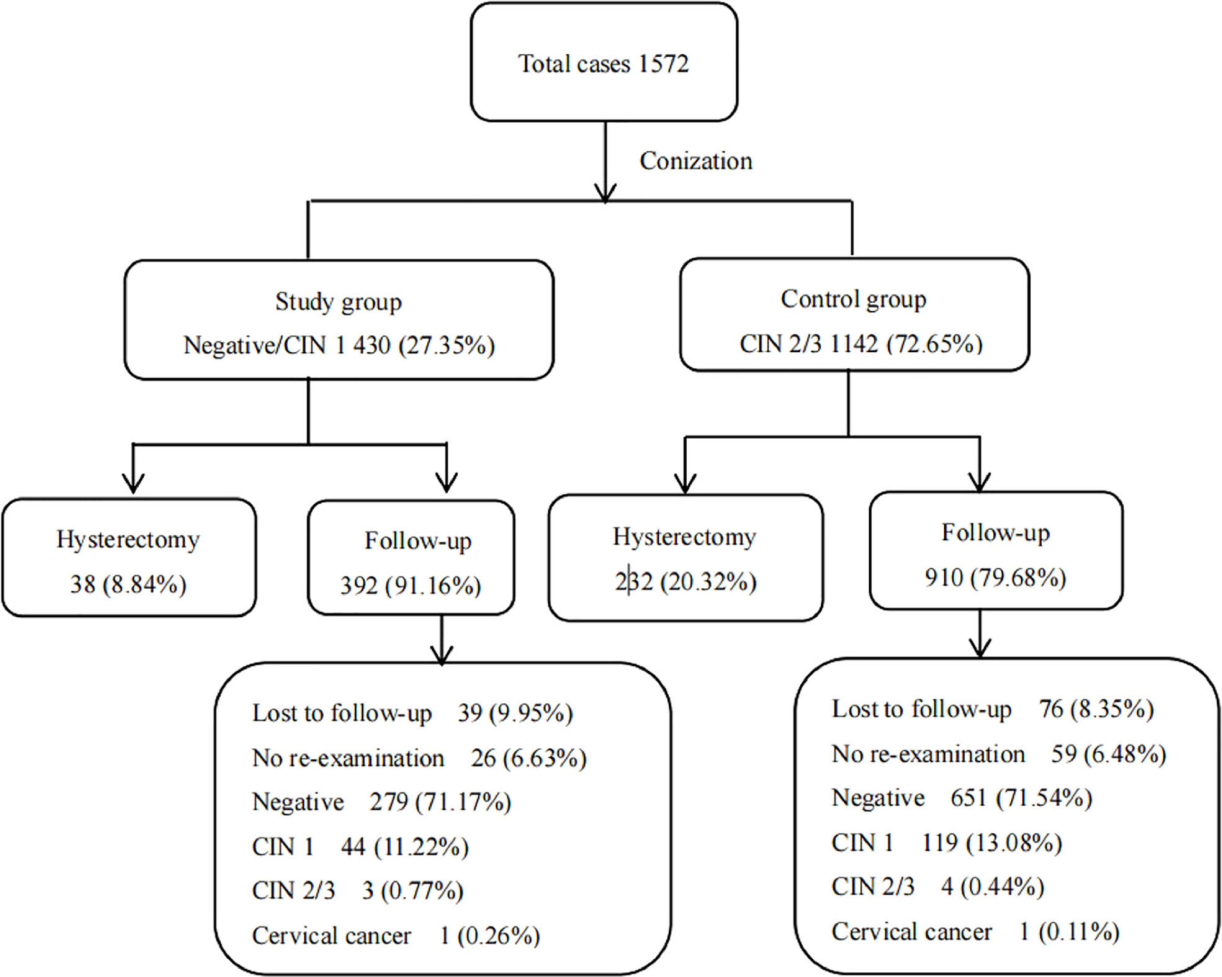
Results of diagnosis and follow-up for 1572 patients.

Of the 430 patients in the study group, 38 patients accepted to undergo hysterectomy, while the other 392 patients were advised to regularly visit a hospital. Of the 1142 patients in the control group, 232 patients accepted to undergo hysterectomy, while 910 patients were advised to regularly visit a hospital (**Figure 1**). We analyzed the follow-up results for the patients who were advised to regularly visit a hospital. The follow-up time for the 1302 patients ranged from 3 months to 108 months, and the median follow-up time was 27 months.

In the study group, 39 patients were lost to follow-up, 26 patients did not visit a hospital, and 327 patients were regularly re-examined, including 279 patients with normal re-examination results, 44 patients with persistent/recurrent CIN 1, three patients with persistent/recurrent CIN 2/3, and one patient with persistent/recurrent cervical cancer. In the control group, 76 patients were lost to follow-up, 59 patients did not visit a hospital, and 775 patients were regularly re-examined, including 651 patients with normal re-examination results, 119 patients with persistent/recurrent CIN 1, four patients with persistent/recurrent CIN 2/3, and one patient with persistent/recurrent cervical cancer (**Figure 1**).

We further compared the persistent/recurrent rates of the two groups. For the study group, the rates of persistent/recurrent CIN1 and persistent/recurrent CIN 2/3 and above were 13.46% (44/327) and 1.22% (4/327), respectively. For the control group, the rates of persistent/recurrent CIN 1 and persistent/recurrent CIN 2/3 and above were 15.35% (119/775) and 0.65% (5/775), respectively. We found that there were no significant differences in terms of the rates of persistent/recurrent disease after conization between patients in the study and control groups (*x^2^* = 1.55, *P* = 0.46).

## Discussion

In our study, a significant percentage of patients (27.35%) initially diagnosed with CIN 2/3 were shown to have either no lesion or a grade of only CIN 1 after thorough examination of a conization specimen. Previous research reported that the corresponding rates ranged from 16% to 25% (9, 12–14). The differences were related to conization indications and the definition of pathological discrepancy. This study defined pathological discrepancy as a diagnosis of CIN 2/3 on colposcopic biopsy and the identification of a grade of CIN 1 or less in conization specimens as negative conization. Most studies identified CIN 1 as positive conization.

Cases of biopsy-confirmed CIN 2/3 with an absence of high-grade dysplasia in conization specimens have diverse interpretations. First, small cervical lesions may have been completely removed by the biopsy. This is the most common cause of negative conization (17). Second, spontaneous regression of the residual lesion after punch biopsy may occur. It has been estimated that spontaneous regression of CIN 2/3 after a confirmatory biopsy occurs in up to 20% of cases (18), especially in young women and when the time interval between biopsy and conization is longer (19). In our study, 97.71% of conizations were performed within 3 months after biopsy, with the exception of a small number of referral patients. Third, the operator was inexperienced, and in cases of lesions located deep in the cervical canal or cervical ectropion, conization did not remove the lesion (9). This situation is unlikely in the case of experienced operators. In our study, the procedure was performed by two designated senior physicians. Finally, pathological underdiagnosis is very unlikely in clinical practice because the routine processing of a conization specimen implies the study of all tissue removed, including multiple sections.

In summary, the most likely reason for negative conization in this study is that the lesions had been completely removed by the biopsy. In addition, there is also a small probability that the lesions had regressed. Both possibilities raise concerns about overtreatment. We further analyzed possible relative factors of negative conization to investigate whether preoperative predictors might reduce unnecessary conization. After univariate and multivariate logistic regression, it was found that patients aged 18–24 years; with a negative hrHPV test result; a cytology test result of normal, ASC, or LSIL; an indication of CIN 2 on pre-conization biopsy; and no glandular involvement were more likely to have an absence of high-grade dysplasia in conization specimens.

Young women needed particular attention because conization could lead to cervical insufficiency. Previous studies have not found an association between age and an absence of high-grade dysplasia in conization specimens (13, 14, 20), which was possibly due to limited sample sizes and a lack of details regarding age groups. In this study, we found that patients aged 18–24 years were more likely to have an absence of high-grade dysplasia in conization specimens. The main reasons for this situation may be that young patients are often found to have satisfactory colposcopy findings, the probability of single-focal lesions is high, and lesions are more likely to be completely removed during colposcopy. In addition, spontaneous regression of CIN 2/3 after a confirmatory biopsy was more likely to occur in young women. McAllum et al. (21) conducted a retrospective review that included women aged < 25 years old with biopsy-proven CIN 2 and found that 98 women (62%) who were managed conservatively showed spontaneous regression.

Results of preoperative hrHPV and cytology testing were also very important predictors. Some studies have found that a preoperative negative hrHPV test result or a low viral load is associated with negative conization. Walavalkar et al. (22) found that patients with negative conization were hrHPV-negative. Ryu et al. (20) reported that a low HPV load (< 100 RLUs) was significantly closely associated with an absence of dysplasia in LEEP specimens. A study by Rodriguez-Manfredi et al. (12) showed that a negative pre-conization hrHPV test result or a low viral load (< 10 RLUs) significantly increased the probability of absence of CIN in conization specimens (75.0% vs. 52%, respectively) in comparison with patients with a high viral load (26.7%; *p* < 0.001). In this study, we found that, in comparison with hrHPV-positive patients, hrHPV-negative patients were more likely to have an absence of high-grade dysplasia in conization specimens. However, this was a retrospective study, and the hrHPV detection methods were not consistent. Thus, there was no method of performing a more accurate analysis of the HPV load, resulting in no relevant conclusions.

Our study also identified that a cytology result of normal, ASC-US, LSIL, or ASC-H was a significant relative factor for an absence of high-grade dysplasia in conization specimens. In previous studies, correlations between cytology findings and negative conization were also found. Walavalkar et al. (22) reported that conization based on cell abnormalities was associated with a higher proportion of negative results (37% for HSIL, 46% for ASC-H, and 76% for LSIL). Rodriguez-Manfredi et al. (12) found that patients with minor abnormalities on pre-conization colposcopy examination had an increased probability of having no lesions in conization specimens. Poomtavorn et al. (14) reported that a low-grade Pap test result (a low-grade Pap test result included ASC-US and LSIL; a high-grade Pap test result included HSIL, ASC-H, AGC, and cancer) was a predictor of negative conization, with an OR of 6.410 (2.877, 14.280). We need to pay close attention to patients with preoperative negative hrHPV or non-HSIL cytology test results.

We found that an indication of CIN 2 on pre-conization biopsy and an absence of glandular involvement were also independent relative factors. Previous studies found that CIN 2 in colposcopically directed biopsy specimens was a predicting factor for having CIN 1 or less in LEEP specimens (11, 23, 24). However, glandular involvement has not been found to be associated with negative conization in previous studies. We speculated that in HSIL, the involvement of glands implies that the lesion site involves crypts and is thus deeper than non-glandular involvement. The lesion severity, number of glands involved, and depth of the location increasing with an increase in the CIN level. In contrast, the presence of non-glandular involvement implies that the lesions are relatively slight. Therefore, the probability of negative conization increases.

We further analyzed the follow-up data and found that there was no difference in the postoperative persistence/recurrence rate between the two groups, which was consistent with previous studies (9, 12, 25). Kyehyun Nam et al. (9) found that a persistent/recurrent disease of CIN 2 grade or worse developed in 3.3% (3/90) of patients with no dysplasia in LEEP specimens, similar to the 5.2% (22/421) of patients with dysplastic lesions in LEEP specimens. Rodriguez-Manfredi et al. (12) also found that no significant differences were observed in terms of percentage of persistent/recurrent disease after conization between patients from the study and control groups. Therefore, for patients with an absence of high-grade dysplasia in conization specimens, we should also emphasize the importance of close follow-up, and the frequency of follow-up should not be reduced merely because conization results are negative.

## Conclusions

We retrospectively analyzed the clinical characteristics of 1695 patients who underwent conization for CIN 2/3 in the past 9 years and found that 430 patients (27.35%) had a grade of CIN 1 or less in conization specimens. An age of 18–24 years, a negative hrHPV test result, a non-HSIL cytology test result, an indication of CIN 2 on pre-conization biopsy, and an absence of glandular involvement in lesions on colposcopy biopsy were independent relative factors for an absence of high-grade dysplasia in conization specimens. We suggested that for patients with relative factors, especially for young women, informed follow-up should be considered. However, whether the choice is conization or informed follow-up, the frequency of follow-up should not be reduced. This retrospective study has certain limitations, such as inconsistent HPV detection methods and incomplete colposcopy data. If these data are available, an in-depth analysis of the results will be more meaningful.

## Data availability statement

The original contributions presented in the study are included in the article/**Supplementary Material**, further inquiries can be directed to the corresponding authors.

## Author contributions

XW and QM conceived and designed the research. YG and YW designed the methods and analyzed the data. YG, YW, and QP contributed to the manuscript writing and interpretation. LL, MZ, and CW participated in the investigations. All authors contributed to the article and approved the submitted version.

## Funding

This work was supported by the Training Program for Young and Middle-aged Medical Talents of Wuhan and Maternal and Child Health Hospital of Hubei Province (No. 220940014).

## Conflict of interest

The authors declare that the research was conducted in the absence of any commercial or financial relationships that could be construed as a potential conflict of interest.

## Publisher’s note

All claims expressed in this article are solely those of the authors and do not necessarily represent those of their affiliated organizations, or those of the publisher, the editors and the reviewers. Any product that may be evaluated in this article, or claim that may be made by its manufacturer, is not guaranteed or endorsed by the publisher.
